# Effects of prostratin on Cyclin T1/P-TEFb function and the gene expression profile in primary resting CD4^+ ^T cells

**DOI:** 10.1186/1742-4690-3-66

**Published:** 2006-10-02

**Authors:** Tzu-Ling Sung, Andrew P Rice

**Affiliations:** 1 Department of Molecular Virology and Microbiology, Baylor College of Medicine, Houston, Texas 77030, USA

## Abstract

**Background:**

The latent reservoir of human immunodeficiency virus type 1 (HIV-1) in resting CD4^+ ^T cells is a major obstacle to the clearance of infection by highly active antiretroviral therapy (HAART). Recent studies have focused on searches for adjuvant therapies to activate this reservoir under conditions of HAART. Prostratin, a non tumor-promoting phorbol ester, is a candidate for such a strategy. Prostratin has been shown to reactivate latent HIV-1 and Tat-mediated transactivation may play an important role in this process. We examined resting CD4^+ ^T cells from healthy donors to determine if prostratin induces Cyclin T1/P-TEFb, a cellular kinase composed of Cyclin T1 and Cyclin-dependent kinase-9 (CDK9) that mediates Tat function. We also examined effects of prostratin on Cyclin T2a, an alternative regulatory subunit for CDK9, and 7SK snRNA and the HEXIM1 protein, two factors that associate with P-TEFb and repress its kinase activity.

**Results:**

Prostratin up-regulated Cyclin T1 protein expression, modestly induced CDK9 protein expression, and did not affect Cyclin T2a protein expression. Although the kinase activity of CDK9 in vitro was up-regulated by prostratin, we observed a large increase in the association of 7SK snRNA and the HEXIM1 protein with CDK9. Using HIV-1 reporter viruses with and without a functional Tat protein, we found that prostratin stimulation of HIV-1 gene expression appears to require a functional Tat protein. Microarray analyses were performed and several genes related to HIV biology, including APOBEC3B, DEFA1, and S100 calcium-binding protein genes, were found to be regulated by prostratin.

**Conclusion:**

Prostratin induces Cyclin T1 expression and P-TEFb function and this is likely to be involved in prostratin reactivation of latent HIV-1 proviruses. The large increase in association of 7SK and HEXIM1 with P-TEFb following prostratin treatment may reflect a requirement in CD4^+ ^T cells for a precise balance between active and catalytically inactive P-TEFb. Additionally, genes regulated by prostratin were identified that have the potential to regulate HIV-1 replication both positively and negatively.

## Background

A latent HIV-1 reservoir in resting memory CD4^+ ^T cells is a major obstacle to the clearance of infection by HAART. The latently infected cells are quiescent and express little if any viral antigens, making it difficult for the immune system to recognize and extinguish them. Cessation of antiviral drugs almost invariably leads to reactivation of high levels of viral replication from this reservoir. The slow turnover of memory CD4^+ ^T cells contributes to the maintenance of the reservoir, and ongoing virus replication that is below the detection limit may continue to reseed the reservoir in the presence of HAART (reviewed in [1-3]).

Mechanisms that establish HIV latency in infected memory CD4^+ ^T cells are not well understood, but it is likely that multiple mechanisms are involved. It has been proposed that latency can result when HIV-1 infects a CD4^+ ^T cell that has been activated and is returning to a quiescent state as the resting memory CD4^+ ^T cell phenotype is established [1]. Blocks to transcription of latent provirus are likely to involve limiting amounts of cellular factors that are essential for RNA polymerase II transcription directed by the viral long terminal repeat (LTR) sequences, such as NF-κB, NF-AT, and the Cyclin T1/P-TEFb complex that mediates the viral Tat protein function. Additionally, HIV-1 integration in heterochromatin regions of the genome may be a factor in some latent infections [4].

The viral Tat function is likely to be a key component of latency. Cyclin T1/P-TEFb consists of Cyclin T1 and CDK9 which can phosphorylate the carboxyl-terminal domain (CTD) of RNA polymerase II and factors that negatively regulate transcriptional elongation, leading to enhanced transcription processivity. The HIV-1 Tat protein recruits Cyclin T1/P-TEFb to the TAR RNA structure located at the 5' end of viral transcripts to promote transcription elongation of the integrated provirus (reviewed in [5-7]). Cyclin T1/P-TEFb is subject to positive regulation in resting CD4^+ ^T cells, as activation of these cells by phytohaemagglutinin (PHA) or combinations of cytokines induces Cyclin T1/P-TEFb [8]. Cyclin T1/P-TEFb is also subject to negative regulation, as a small nuclear RNA known as 7SK snRNA and the major HEXIM1 and minor HEXIM2 proteins are recently identified P-TEFb-associated factors that repress kinase activity [9,10]. In support of the idea that 7SK and HEXIM1 proteins repress P-TEFb function, depletion of 7SK snRNA by anti-sense DNA oligonulceotides or siRNAs activates transfected reporter plasmids [10,11]. Additionally, over-expression of HEXIM1 can repress Tat activation of an HIV-1 LTR reporter plasmid [12,13]. However, following activation of peripheral blood lymphocytes (PBLs), the association of 7SK snRNA with P-TEFb is greatly increased and this correlates with active RNA polymerase II transcription [14]. We recently observed that while siRNA depletion of 7SK snRNA in HeLa cells stimulates expression of reporter plasmids and induces apoptosis, it does not affect expression of the endogenous P-TEFb-dependent cellular genes or of HIV-1 reporter viruses [11]. Thus, although perturbation of the normal level of association of 7SK and HEXIM1 with P-TEFb can influence expression from transfected plasmids, the effects on endogenous P-TEFb-dependent genes or an integrated HIV-1 provirus are less apparent.

Recent studies have focused on the search for adjuvant therapies that can reactivate the HIV latent reservoir under conditions of HAART to suppress active viral replication, thus reducing the size of the reservoir with the ultimate goal of eradication. Prostratin, a non-tumor-promoting phorbol ester, is a candidate for such an adjuvant strategy [15]. Prostratin has been shown to activate NF-κB and reactivate latent HIV [16-18]. Although prostratin stimulates the expression of T cell activation markers, it does not promote cell proliferation, therefore lowering the risks of expanding the latently-infected cell population [15,19]. The majority of studies of prostratin focused on its role in reactivation of HIV from transcriptional latency [20,21], but prostratin may also affect other stages of virus replication. Indeed, prostratin has been shown to down-regulate CD4 and CXCR4 to inhibit viral entry through PKC pathways and to block reverse transcription [19,22,23]. These dual effects of prostratin, activating latent HIV and inhibiting further spreading of the virus, appear to meet the criteria for a useful adjuvant therapy. To further examine mechanisms involved in reactivation of proviruses by prostratin, we examined effects of prostratin on P-TEFb in resting CD4^+ ^T cells isolated from healthy blood donors. We also carried out a transcriptional profile analysis to identified genes of relevance to HIV infection that are regulated by prostratin.

## Results

### Prostratin up-regulates Cyclin T1 but not Cyclin T2a in resting CD4^+ ^T cells

**Figure 1:**
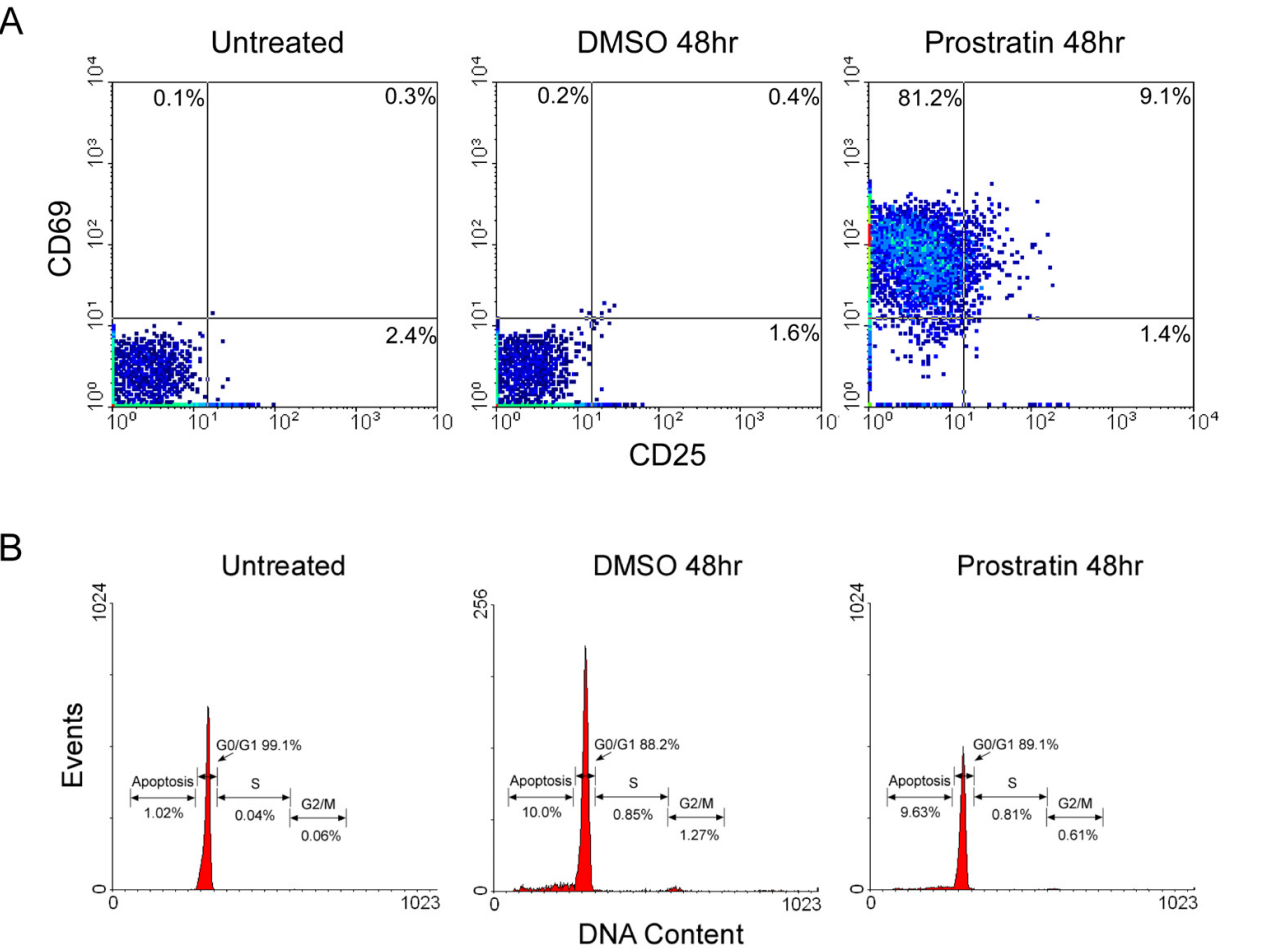
**Prostratin induces expression of CD69 without promoting cell cycle progression in resting CD4^+ ^T cells**. Resting CD4^+ ^T cells were analyzed immediately after isolation (Untreated), or were cultured for 48 hours in the presence of DMSO as a control or prostratin (250 ng/ml) before analysis. (A) Cells were assayed for expression of CD25 and CD69 by flow cytometry. (B) Cells were stained with propidium iodide to evaluate DNA content by flow cytometry.

We wished to examine whether the induction of HIV-1 proviral transcription in latently infected cells by prostratin might involve an up-regulation of Cyclin T1/P-TEFb, a mediator of the viral Tat activation function. To confirm that prostratin induced early T cell activation markers without promoting cellular proliferation under our experimental conditions, resting CD4^+ ^T cells isolated from healthy donors were treated with dimethyl sulfoxide (DMSO) as a solvent control or prostratin for 48 hours, and expression of CD25 and CD69 was evaluated by flow cytometry (Fig. 1A). Additionally, a portion of untreated cells were examined immediately after isolation. Prostratin treatment induced expression of CD69, and had a modest increase on CD25 expression. Propidium iodide staining was also performed to evaluate cell cycle progression (Fig. 1B). Prostratin had no effect on cellular proliferation, as the percentage of cells in S and G2/M phases was similar in prostratin-treated and control cells. In addition, apoptosis appeared to occur in approximately 10% of cells in both control and prostratin-treated cells. We conclude that under these conditions, prostratin induces expression of CD69 and to a limited extent CD25, but does not induce cellular proliferation nor enhance apoptosis, in agreement with previous studies [18,19].

**Figure 2:**
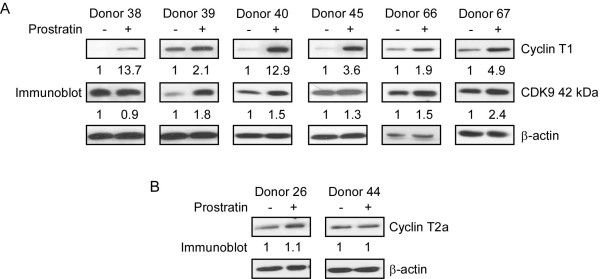
**Effects of prostratin on the expression levels of Cyclin T1, Cyclin T2a, and CDK9**. Cell extracts were prepared from resting CD4^+ ^T cells from different donors cultured in DMSO or prostratin for 48 hours, and immunoblots were performed to examine the levels of Cyclin T1, CDK9, β-actin (A) and Cyclin T2a (B). The immunoblots were quantified as described in Material and Methods using β-actin for normalization; the value for fold-induction is given below the panels.

We and others have reported that activation of PBLs and resting CD4^+ ^T cells with PHA, a lectin mitogen, induces cell proliferation and the expression of Cyclin T1 and CDK9, components of the Cyclin T1/P-TEFb complex [8,24-27]. To examine whether prostratin induces Cyclin T1 and CDK9, immunoblots were performed with extracts prepared from cells treated for 48 hours with DMSO or prostratin. We examined resting CD4^+ ^T cells isolated from a number of donors, and results from six representative donors are shown in Fig. 2A. The levels of β-actin, a loading control, were equivalent in each extract. After prostratin treatment, Cyclin T1 level increased in Donors 38, 40, and 45 from almost undetectable levels in control cells to levels of induction ranging from approximately four- to 14-fold after normalization to β-actin levels. For Donors 39, 66, and 67, Cyclin T1 was expressed at a basal level in the resting cells and was induced from two- to five-fold by prostratin. In contrast to Cyclin T1, the major CDK9 42 kDa protein was present at readily detectable levels in control cells and was not further up-regulated in Donors 38 and 45, while in Donors 39, 40, 66, and 67, CDK9 levels increased from 1.5- to 2.5-fold following prostratin treatment. We observed that the 55 kDa minor form of CDK9 was generally presented at low levels in resting CD4^+ ^T cells and in a few donors it was induced approximately two-fold (data not shown).

We also examined the levels of Cyclins T2a and T2b, two additional cyclin partners of CDK9. Cyclin T2a expression was not significantly affected by prostratin (Fig. 2B), while the less abundant Cyclin T2b was below the level of detection in our system (data not shown). We conclude from these experiments that prostratin up-regulates Cyclin T1 expression and has a relatively modest stimulatory effect on CDK9 expression levels in resting CD4^+ ^T cells. Because expression of Cyclin T2a was not affected by prostratin activation, this P-TEFb regulatory subunit may be generally involved in constitutive gene expression in resting and activated CD4+ T cells, whereas Cyclin T1 may play a larger role in the expression of genes induced by T cell activation.

### Effects of prostratin on the levels of 7SK snRNA and HEXIM1 associated with CDK9

**Figure 3:**
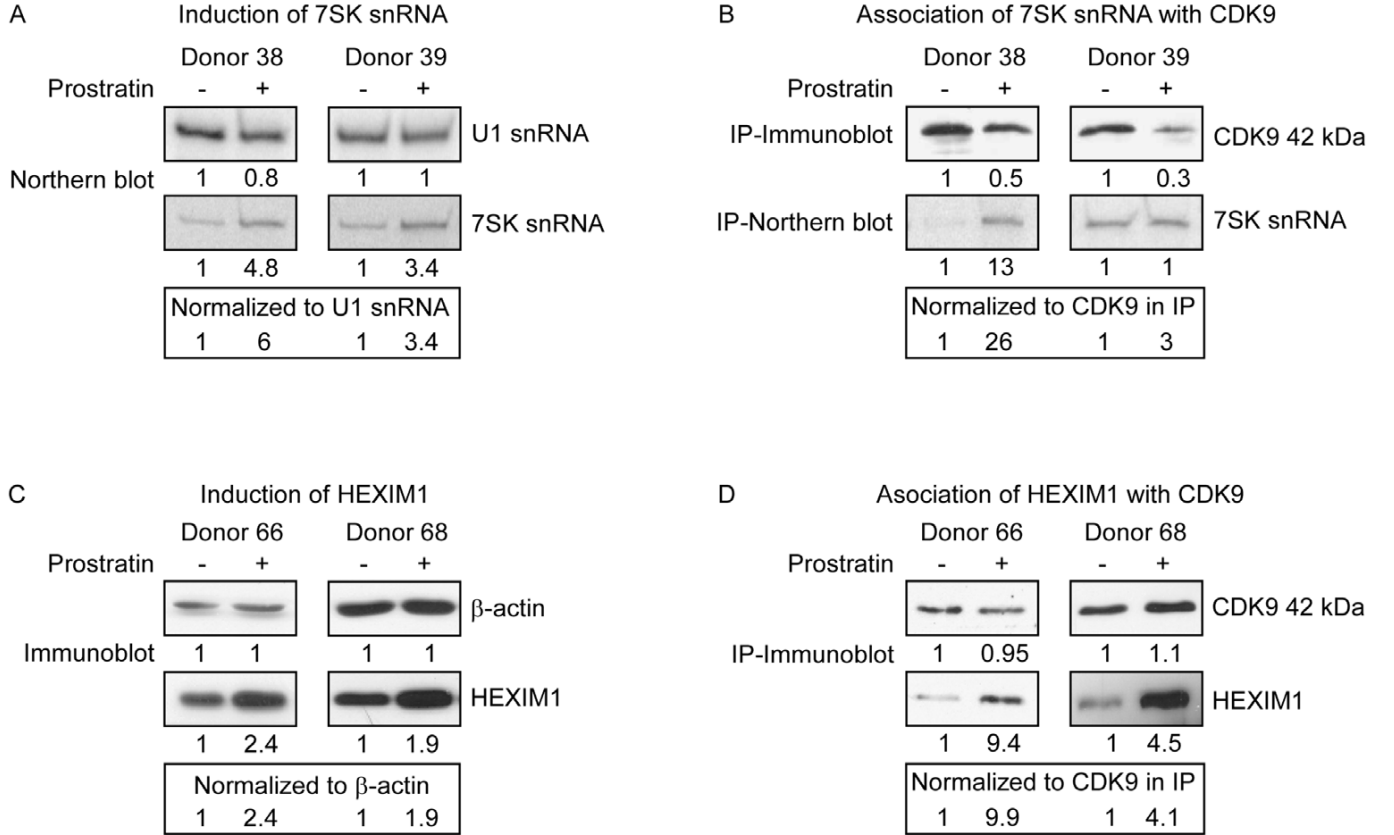
**Levels of 7SK snRNA and HEXIM1 and association with P-TEFb**. (A) Total RNA was isolated from DMSO control and prostratin-treated resting CD4^+ ^T cells. Northern blots were performed to measure 7SK snRNA and U1 snRNA levels; amounts of 7SK and U1 snRNA were quantified using a Phosphoimager and are shown below each panel. Levels of 7SK snRNA were normalized to U1 snRNA. (B) Immunoprecipitations were performed using α-CDK9 antibodies with extracts from control and prostratin-treated cells. CDK9 levels present in a portion of immunoprecipitates were examined by immunoblots (IP-Immunoblot). RNA was extracted from the remaining protein of immunoprecipitates and the levels of 7SK snRNA were determined by Northern blots (IP-Northern blot). Amounts of 7SK snRNA were quantified by a PhosphoImager and normalized the amounts of CDK9 present in immunoprecipitates. (C) Cell extracts were prepared from resting CD4^+ ^T cells cultured in DMSO or prostratin for 48 hours to examine the levels of HEXIM1 and β-actin. Protein levels were quantified by the Densitometer and are shown below each panel. Levels of HEXIM1 were normalized to β-actin levels. (D) Immunoprecipitations were performed using antiserum against CDK9 with extracts adjusted to precipitate equivalent amount of CDK9. Immunoprecipitation products were subjected to immunoblot analysis to evaluate the levels of HEXIM1 associated with CDK9. Levels of HEXIM1 were normalized to CDK9 levels in immunoprecipitates.

We next wished to examine if 7SK snRNA and HEXIM1, two molecules which are known to associate with P-TEFb and repress catalytic activity *in vitro*, are affected by prostratin treatment. For detection of total 7SK levels, we carried out Northern blots of total RNA isolated from control and prostratin-treated cells (Fig. 3A). Total 7SK snRNA levels were increased in prostratin-treated cells compared to control cells, while U1 snRNA remained constant. When normalized to U1 RNA, total 7SK snRNA increased 3.4- and 6-fold in the two donors examined. To evaluate the amount of 7SK associated with P-TEFb, we immunoprecipitated CDK9 and measured 7SK levels in precipitates by Northern blots (Fig. 3B). Although there was a considerable variation between the two donors examined, the association between 7SK snRNA and CDK9 increased significantly after prostratin treatment when normalized to the amount of CDK9 protein in immunoprecipitates, consistent with our previous findings in activated PBLs [14].

We also examined HEXIM1 protein expression levels and its association with CDK9. HEXIM1 was readily detectable in control cells and prostratin treatment induced its expression approximately two-fold in both donors (Fig. 3C). To examined the amount of HEXIM1 associated with P-TEFb, co-immunoprecipitations were performed with antibodies against CDK9 (Fig. 3D). Similar to 7SK snRNA, the levels of HEXIM1 associated with CDK9 increased in prostratin-treated cell. After normalization to the amount of CDK9 present in immunoprecipitates, this increase was an approximate 9-fold induction in Donor 66 and a 4-fold induction in Donor 68. These data are consistent with previous studies which demonstrated that 7SK snRNA enhances binding of HEXIM1 to P-TEFb [9,28]. Furthermore, these data indicate that prostratin treatment leads to a large increase in the proportion of CDK9 molecules that are associated with 7SK and HEXIM1. However, this increase does not appear to result in a general repression of gene expression, as the cells are responding to a program of T cell activation (see Fig. 1).

### Prostratin increases CDK9 kinase activity in resting CD4^+ ^T cells

**Figure 4:**
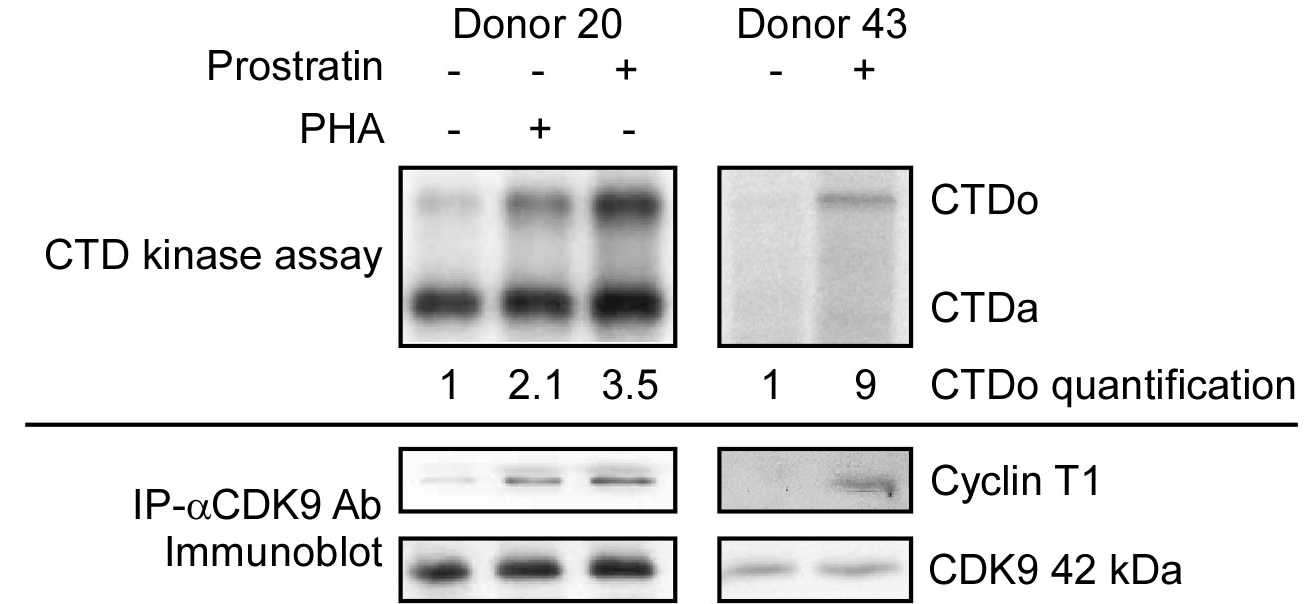
**Effects of prostratin on CDK9 kinase activity**. The amount of cell extracts from DMSO and prostratin-treated cells used in immunoprecipitations were adjusted to precipitate equivalent amount of CDK9 using antiserum against CDK9. Immunoprecipitates were subjected to CTD kinase assays to examine relative kinase activities. Products of kinase assay were examined on SDS-polyacrylamide gels, and the CTD substrate hyperphosphorylated form (CTDo) and hypophosphorylated form (CTDa) are shown at the top. PHA-treated cells were used as a positive control. A portion of immunoprecipitates were analyzed in immunoblots shown at the bottom to confirm that equivalent amounts of CDK9 were precipitated; Cyclin T1 levels present in immunoblots were also evaluated by immunoblots.

Cell activation by a combination of cytokines or PHA has been shown to increase P-TEFb catalytic activity in PBLs and purified resting CD4^+ ^T cells [8,24,26]. Induction of kinase activity by those treatments correlates with increased protein levels for both Cyclin T1 and CDK9. We performed kinase assay with a recombinant CTD substrate to examine if prostratin increases P-TEFb activity in resting CD4^+ ^T cells. Antibodies against CDK9 were chosen for immunoprecipitation (IP) due to the low level of Cyclin T1 in resting cells from some donors. Because the levels of CDK9 can be higher in prostratin-treated cells compared to control cells (see Fig. 2A), amounts of cell extracts used in immunoprecipitation were adjusted so that equivalent amounts of CDK9 would be precipitated and kinase activities would therefore be normalized to CDK9 levels. As shown in Figure 4, equivalent amounts of CDK9 were immunoprecipitated from control and prostratin-treated cell extracts under these conditions. Additionally, we examined the levels of Cyclin T1 that were associated with CDK9. The levels of Cyclin T1 that were co-immunoprecipitated with CDK9 were significantly higher in prostratin-treated cells, likely the result of the low levels of Cyclin T1 in control extracts. Phosphorylation of the CTDo (hyperphosphorylated form) substrate in kinase reactions was significantly higher in immunoprecipitates from prostratin-treated cells than from control cells. PHA-treated cells were used as a positive control and to show the relative positions of the CTDo and CTDa (hypophosphorylated form) substrates. Since equal amounts of CDK9 were precipitated, the data in Fig. 4 indicate that prostratin not only up-regulates protein levels of Cyclin T1 and to some extent CDK9, but it also up-regulates CDK9 kinase activity, which is likely to contribute to the level of transcriptional elongation in prostratin-treated cells. The amounts of resting and prostratin-treated CD4^+ ^T cells that can be obtained for use in biochemical experiments are limited. We were therefore unable to carry out more detailed studies on the effects of prostratin on P-TEFb function as regulated by 7SK snRNA, HEXIM1, and Brd4, a recently identified positive mediator of P-TEFb function [29,30].

### HIV-1 Tat function is likely to be important in prostratin stimulation of viral gene expression

**Figure 5:**
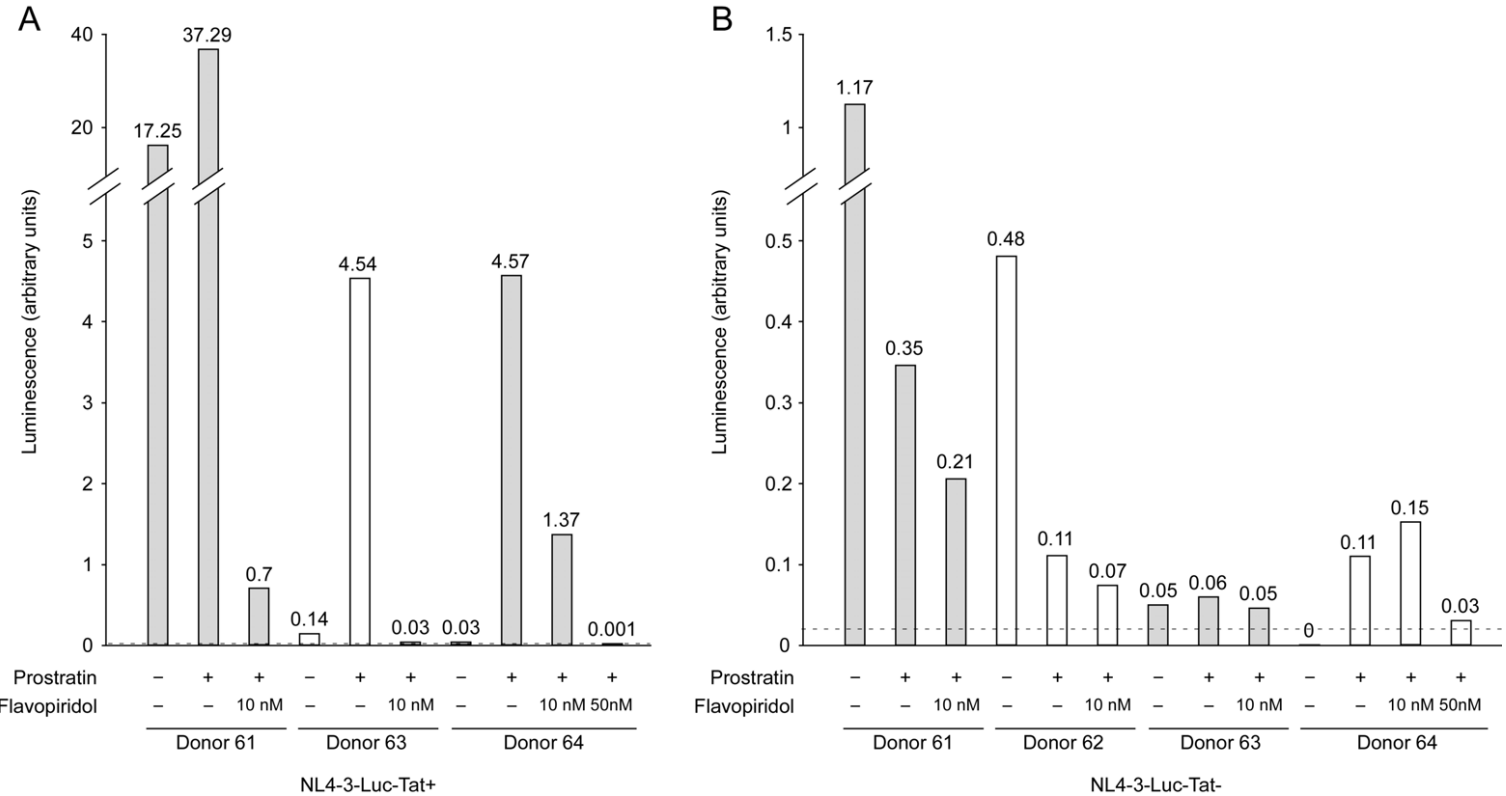
**Cyclin T1/P-TEFb is likely to be important for prostratin stimulation of HIV reporter virus expression**. Resting CD4^+ ^T cells were infected with wild type HIV NL4-3-Luc-Tat^+ ^luciferase reporter virus (A) or NL4-3-Luc-Tat^- ^(B), a mutant reporter virus with a non-functional Tat. After overnight incubation, cells were washed and cultured with DMSO or prostratin. Flavopiridol, a selective P-TEFb inhibitor, was added as indicated simultaneously with prostratin. Cells were harvest 48 hours after prostratin/flavopiridol treatment and reporter plasmid expression was examined by luciferase assays. Dashed lines indicate the background signal in the luciferase assay (~0.025) as determined from the signal in uninfected cell extract.

The data presented in Figure 4 showed that prostratin enhances CDK9 kinase activity, and this may be utilized by the HIV-1 Tat protein to activate expression of the integrated provirus. To examine this possibility, an HIV-1 luciferase reporter virus (NL4-3-Luc-Tat^+^) was used to infect resting CD4^+ ^T cells. After overnight incubation with virus, cells were washed and cultured in the presence of DMSO or prostratin. Additionally, flavopiridol, a selective inhibitor of P-TEFb and therefore Tat function [31], was added to some of the cultures at the time of prostratin treatment. A concentration at 10 nM of flavopiridol was chosen based on previous studies showing sufficient inhibitory effects of P-TEFb activities without significant cytotoxicity at this concentration [31,32]. Cells lysates were prepared 48 hours after prostratin/flavopiridol treatment and luciferase activity was measured to determine reporter virus gene expression. At the time of preparation of extracts, no significant difference in cell viability was observed in cultures treated with flavopiridol. As shown in Figure 5A, prostratin treatment enhanced HIV-1 gene expression, from a 2-fold induction in Donor 61 to large increases in Donor 63 and 64, where fold-activation could not be quantified due to the low background levels of luciferase expression in control infected cells. Addition of flavopiridol at a concentration of 10 nM antagonized the effects of prostratin, resulting in 70% or greater reduction in all three donors. Increasing the flavopiridol concentration to 50 nM resulted in an even greater reduction in HIV-1 gene expression.

To determine if the prostratin enhancement of HIV-1 gene expression is likely to be dependent upon the viral Tat protein, a mutant reporter virus encoding a non-functional Tat protein (NL4-3-Luc-Tat^-^) was used to infect resting CD4^+ ^T cell. In contrast to the virus expressing a functional Tat protein, the Tat^- ^virus infections did not show a significant stimulatory effect when treated with prostratin. In Donors 61 and 62, prostratin treatment actually decreased luciferase activities (Fig. 5B). Luciferase expressions in control cells from Donors 63 and 64 were near background levels (indicated by dashed lines) of the assay and prostratin had a small stimulatory effect whose significance is uncertain. Adding flavopiridol at 10 nM also had variable effects on luciferase expression in the Tat^- ^virus. These data indicate that in the absence of Tat, prostratin has small and variable effects on reporter virus expression, consistent with the proposal that the prostratin induction of Cyclin T1/P-TEFb plays a role in the stimulation of the NL4-3-Luc-Tat^+ ^virus. We note that it is possible that prostratin may also affect steps in the virus life cycle prior to transcription of the integrated provirus, such as reverse transcription or integration. Additionally, the data with the Tat^- ^virus suggest that prostratin might induce cellular factors that negatively affect the HIV-1 gene expression, potentially acting at a stage from uncoating of the virion through translation of luciferase mRNA.

### Prostratin microarray analysis

Prostratin appears to induce both negative and positive functions for HIV-1 gene expression as inferred from infections with the Tat^+ ^and Tat^- ^reporter viruses. We therefore wished to investigate the global effects of prostratin on cellular gene expression. To identify genes affected by prostratin, RNA was isolated from resting CD4^+ ^T cells cultured in the presence of DMSO or prostratin for 48 hours, a time of treatment which had no observable effect on cellular proliferation or apoptosis (Fig. 1B). Gene expression profiles were examined using the Affymetrix GeneChip Human Genome U133 PLUS 2.0 array, which contains about 54,000 probe sets representing approximately 21,000 human genes. Three biological replicates from 3 donors (Donor 32, 33, and 44) were prepared from both DMSO- and prostratin-treated cells, and the data were analyzed by the GeneSifter microarray data analysis system. The analyzed microarray data can be downloaded from Herrmann-Rice laboratory website [33].

We identified a total of 3094 probe sets that are significantly affected by prostratin treatment using filtering criteria of ≥ 1.5 fold-change in expression, the method of Benjamini and Hochberg for multiple testing correction [34], and an adjusted p-value < 0.05. We found that 983 non-redundant transcripts were up-regulated ≥ 1.5-fold and 1531 non-redundant transcripts were down-regulated ≥ 1.5-fold by prostratin. A detailed analysis of the microarray data is presented as Supplemental Data (see Additional files 1, 2, 3, 4).

**Figure 6:**
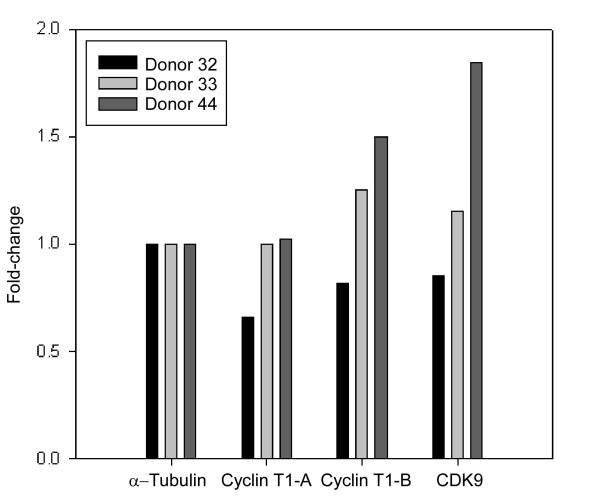
An aliquot of RNA from the three donors for microarray analysis were reverse transcribed for quantitative real-time PCR assays for Cyclin T1, CDK9, and control α-tubulin mRNAs. Two sets of primers were designed to amplify different regions of Cyclin T1 mRNA (see Methods). Fold-change was calculated as the change in transcript levels in prostratin-treated cells relative to DMSO-treated cells after normalization to α-Tubulin levels.

Interestingly, our statistical analysis of the microarray data indicated that the mRNAs for Cyclin T1 and CDK9 were not significantly affected by prostratin treatment. To verify this microarray data, we performed reverse transcription followed by quantitative real-time PCR; in the case of Cyclin T1, we used two sets of primers for the real-time PCR (Fig. 6). The data for Cyclin T1 with primer set A (Cyclin T1-A) showed no induction by prostratin; primer set B (Cyclin T1-B) showed a < 1.5-fold induction in Donors 32 and 33 and a 1.5-fold induction in Donor 44. These data are consistent with the microarray data and suggest that there is less than a 1.5-fold stimulation of Cyclin T1 mRNA by prostratin. The real-time PCR data for CDK9 is somewhat variable between the three donors, but they are consistent with the statistical analysis of the microarray data which indicated that there is not a statistically significant induction of CDK9 mRNA that is ≥ 1.5-fold. Quantitative RT-PCR for other selected mRNAs indicated that the microarray data are in general reliable (see Additional file 2).

Genes with a ≥ 5-fold change by prostratin were identified, and those with relevance to T cell or HIV biology are listed in Table 1. In agreement with GO and KEGG pathway analyses (see Additional files 3 and 4), most of the genes related to T cell biology are involved in cellular activation or apoptosis. The transcripts of CD25 and CD69 were up-regulated > 5-fold by prostratin, indicating the increased protein levels detected by flow cytometry involves transcriptional inductions (Fig. 1A). Expression of LKLF transcript was down-regulated, consistent with its role in regulating T cell quiescence [35,36]. Of relevance to HIV biology, IL7R (interleukin-7 receptor) mRNA level was up-regulated by prostratin, which may affect HIV pathogenesis. Signaling via the IL7R is essential for T cell homeostasis and maintenance of T cell memory, and down-regulation of IL7R correlates with depletion of CD4^+ ^T cells and AIDS (acquired immune deficiency syndrome) progression [37,38]. Interestingly, genes identified in our analysis have conflicting effects on HIV replication. Two up-regulated genes, APOBEC3B and TNFSF4, have been shown to have negative and positive effects on HIV replication, respectively. APOBEC3B is able to suppress the infectivity of HIV-1 [39], while stimulation of TNFSF4 (tumor necrosis factor receptor superfamily, member 4) by its ligand enhances HIV-1 infection [40]. DEFA1, the most highly down-regulated gene in our analysis (24-fold), has been reported to have anti-HIV activity involving steps following reverse transcription and integration [41]. The S100 calcium-binding protein transcripts (S100A8, 9, 12), which were down-regulated by prostratin, induce HIV-1 transcriptional activity and viral replication in infected CD4^+ ^T lymphocytes [42]. These observations that prostratin affects cellular mRNAs with both positive and negative effects on HIV-1 replication suggest that the net effect of prostratin on HIV-1 infection of CD4^+ ^T cells may reflect a balance of different gene functions, including stimulation of Tat function by the induction of Cyclin T1/P-TEFb activity.

**Table 1:** Genes relevant to HIV or T cell biology

	**Direction**	**Gene ID**	**Relevance**
**HIV biology**	Up	APOBEC3B	Anti-HIV-1 activity [39]
		CCL3	An HIV-suppressive factor produced by activated CD8^+ ^T cells [59]
		EGR1,2	HIV-1 Tat binds EGRs and induces FasL up-regulation [60]
		HIVEP3	A zinc finger protein regulating transcription via the kappa-B enhancer motif [61]
		IL7R	Correlates with CD4^+ ^T cell depletion in HIV-infected patients [37]
		TNFSF4	Enhances HIV-1 replication [40]

	Down	DEFA1	Inhibits HIV-1 replication [41]
		S100A8, 9, 12	Induce HIV-1 transcription and replication [42]

**T cell biology**	Up	CD25, 69, 96	Cell activation markers
		DUSP4,5,6,10	Involved in MAPK pathway [62–65]
		PMAIP1	Involved in p53-mediated apoptosis [66]
		TNFRSF9	Inhibits proliferation of activated T lymphocytes, induces programmed cell death [67]

	Down	LKLF	T cell quiescence [35]
		LIME1	Involved in T cell activation [68]
		GILZ	Involved in T cell activation, anti-inflammatory and immunosuppressive effects [69, 70]

## Discussion

In this study, we found that prostratin up-regulated Cyclin T1 protein expression and had a modest induction on CDK9 protein expression. The induction of Cyclin T1 may involve post-transcriptional mechanisms, as Cyclin T1 mRNA levels were not significantly induced by prostratin. The increased Cyclin T1 protein expression by prostratin is likely to be a main cause of the increased association of Cyclin T1 with CDK9 as measured by co-immunoprecipitation (Fig. 4). The expression of Cyclin T2a, another Cyclin partner of CDK9, remained largely unchanged by prostratin (Fig. 2B), suggesting the presence of Cyclin T2a does not prevent the induced Cyclin T1 from binding to CDK9. The increased association of Cyclin T1 with CDK9 leads to elevated Cyclin T1/P-TEFb kinase activity, and this appears to be utilized by the HIV-1 Tat protein to stimulate viral LTR-directed transcription as suggested by our results with Tat^+ ^reporter virus infection (Fig. 5A). Thus, the prostratin reactivation of latent HIV-1 provirus is likely to induce Tat function through an up-regulation of Cyclin T1/P-TEFb.

In the absence of a functional Tat protein, prostratin exhibited variable and somewhat negative effects on virus gene expression (Fig. 4B). It has been shown previously that prostratin inhibits reverse transcription but facilitates proviral integration [19], which may contribute to the variable effects of prostratin in the absence of Tat when viral gene expression is low. The overall outcome of viral gene expression observed in this study may represents the net result of different effects of prostratin, among which the Tat-mediated transactivation through Cyclin T1/P-TEFb plays a major role. Additionally, P-TEFb has been shown to bind to NF-κB and contribute to stimulation of elongation by this transcription factor [43]. Thus, prostratin stimulation of NF-κB through the up-regulation of Cyclin T1/P-TEFb is also likely to contribute to stimulation of viral gene expression [44].

The expression levels of 7SK snRNA and HEXIM1 protein, two negative regulators of P-TEFb, were also induced by prostratin treatment, and this lead to a large increase in the proportion of CDK9 molecules that were associated with 7SK and HEXIM1 (Fig. 3). These observations indicate that a large increase in the association of 7SK/HEXIM1 with P-TEFb does not generally repress gene expression in CD4^+ ^T cells, consistent with our previous studies in PBLs [14]. These data are not inherently in disagreement with 7SK/HEXIM1 acting as a negative regulator of P-TEFb. In resting CD4^+ ^T cells, only low levels of transcriptional elongation are needed and the levels of P-TEFb are low. Upon T cell activation, there is an increase in the overall level of P-TEFb to fulfill the requirements for the transcriptional program of T cell activation. With higher overall levels of P-TEFb, it may be necessary to increase the levels of 7SK and HEXIM1 to maintain a precise balance between active and inactive P-TEFb. In this scenario, the total level of P-TEFb, both active and inactive in the 7SK/HEXIM1 complex, is always in excess over the transcriptional requirements of the cell. If there is a need for increased transcription, active P-TEFb can be rapidly recruited from the pool of inactive molecules in the 7SK/HEXIM1 complex.

The phosphorylation of CDK9 at threonine-186 in the T-loop is crucial for the association between 7SK snRNA and Cyclin T1/P-TEFb [45]. This phosphorylation is likely to be induced by prostratin and play a key role in the increased association of 7SK snRNA with P-TEFb. Therefore, identifying the kinase responsible for this phosphorylation of CDK9 is important for further insight into this issue. The binding of HEXIM1 to CyclinT1/P-TEFb is known to be dependent on 7SK snRNA, and the carboxyl-terminus of HEXIM1 itself is important for interaction between HEXIM1 and Cyclin T1 [13,28,46]. Although the assembly sequence and signals required for Cyclin T1 P-TEFb/7SK/HEXIM1 associations are complex and inter-dependent, the increased Cyclin T1 levels very likely contribute to the increased association.

### Supplemental data

We performed a comprehensive transcriptional profile analysis with an Affymetrix GeneChip Human Genome U133 PLUS 2.0 array. Several transcripts were selected for validation of the Affymetrix data by reverse transcription followed by quantitative real-time PCR. CD69, dual-specificity phosphatase 4 (DUSP4), and early growth response 1 (EGR1) genes were selected to represent up-regulated transcripts identified in the microarray analysis. Lung Kruppel-like transcription factor (LKLF), defensin α1 (DEFA1), and S100 calcium-binding protein A8 (S100A8) genes were selected to represent down-regulated transcripts. The α-Tubulin gene was selected to represent a transcript that was present but not affected by prostratin treatment for normalization. The results of reverse transcription/real-time PCR assays agreed well with the microarray data in all cases, indicating that the microarray data are in general reliable (see Additional file 2).

To identify the biological processes to which the prostratin-regulated genes belong, predominant functional themes were mapped by GeneSifter on the Gene Ontology (GO) hierarchy in combination with Cytoscape using BiNGO (see Methods). To generate a hierarchic illustration of the GO categories in biological process, non-redundant gene lists generated by GeneSifter and modified by removal of redundant genes were analyzed by BiNGO. Additional file 3 illustrates the GO categories that were over-represented among genes up-regulated by prostratin treatment in resting CD4^+ ^T cells. The size of individual nodes is indicative of the numbers of genes involved in the category and the color represents the level of significance, with orange indicating the highest significance. Immune responses and apoptosis-related genes were highly over-represented, consistent with the roles of prostratin in CD4^+ ^T cell activation [47,48]. We noted that "regulation of IκB kinase/NF-κB cascade" was over-represented among up-regulated genes, agreeing well with a previous study which showed that prostratin activates NF-κB [17]. For the biological ontology processes of down-regulated genes shown in Additional file 3, processes related to metabolism, growth, and apoptosis were over-represented, especially processes related to protein modification. Although both pro- and anti-apoptotic genes were over-represented in prostratin-regulated transcripts, it is notable that apoptosis is not enhanced in prostratin-treated cells.

KEGG (Kyoto Encyclopedia of Genes and Genomes) pathway is a collection of pathway maps representing molecular interaction and reaction networks for cellular processes or pathways. Using the KEGG pathway analysis provided by GeneSifter, we identified several pathways that are significantly affected by prostratin with a z-score >2 (see Additional file 4). A z-score >2 is considered to represent a pathway that is over-represented among a given gene list [49]. In agreement with the gene ontology analysis, the apoptotic pathway was over-represented in both up- and down-regulated genes. Pathways related to protein degradation, proteasome and ubiquitin mediated proteolysis were also identified, in agreement with the enriched ontology categories related to protein metabolism shown in Figure 6B. In addition, cytokine-cytokine receptor interaction, MAPK signaling pathway, and phosphatidylinositol signaling system were also identified in the analysis, suggesting that prostratin affects signal transduction pathways.

In our transcriptional profiling and GO analysis, the majority of the over-represented gene categories match expected characteristics of prostratin in resting CD4^+ ^T cell activation, such as death, immune response, and metabolism. Importantly, we identified pathways and genes worth further investigation. KEGG pathway analysis indicated certain prostratin-regulated pathways that have not been examined. The MAPK signaling pathway and the phosphatidylinositol signaling system have been associated with T cell activation, proliferation, and death [50,51], and our observation that prostratin may activate these two pathways provides insight into the effects of prostratin on resting CD4^+ ^T cells.

## Conclusion

We found that prostratin induced expression of Cyclin T1 and P-TEFb function which appears to be utilized by the HIV-1 Tat protein to enhance viral gene expression. Cyclin T2a, an alternative regulatory subunit of P-TEFb, was not induced by prostratin. Prostratin increased expression of 7SK snRNA and HEXIM1 protein and their association with CDK9. Because 7SK and HEXIM1 are negative regulators of P-TEFb, these results suggest that as the overall level of P-TEFb increases in CD4^+ ^T cells, there is a requirement to maintain a precise balance between active P-TEFb and inactive P-TEFb. Using microarray to analyze the global pattern of gene expression, we identified a number of genes of significance to HIV-1 replication, both positive and negative regulators that are affected by prostratin.

## Methods

### Isolation of resting CD4^+ ^T cells

Peripheral blood mononuclear cells (PBMC) were isolated from healthy donors (Gulf Coast Regional Blood Center, Houston, TX) by Isolymph density gradient centrifugation (Gallard-Schlesinger). CD4^+ ^T cells were purified from PBMCs by negative selection with a CD4^+ ^T cell isolation kit II (Miltenyi Biotec) based upon a cocktail of biotin-conjugated antibodies against CD8, CD14, CD16, CD19, CD36, CD56, CD123, TCR γ/δ, and glycosphorin A and α-biotin magnetic microBeads. Purity of CD4^+ ^T cell preparations was between 92 to 98% pure as evaluated by flow cytometry using a Beckman-Coulter XL-MCL cytometer with fluorescein isothiocyanate (FITC)-conjugated α-CD4 antibodies and phycoerythrin (PE)-conjugated α-CD3 antibodies (BD PharMingen). To obtain resting CD4^+ ^T cells, activated cells were further depleted with α-CD30 magnetic microbeads (Miltenyi Biotec).

### Prostratin treatment and propidium iodide (PI) staining

Purified resting CD4^+ ^T cells were cultured in RPMI with 10% fetal bovine serum (FBS) and 1% penicillin/streptomycin and were treated with prostratin (12-deoxyphorbol 13-acetate, kindly provided by Dr. Stephen Brown, AIDS ReSearch Alliance, West Hollywood, CA) at a concentration of 250 ng/ml or DMSO as the solvent control. Cells were harvested at 48 hours after treatment and were washed with phosphate-buffered saline (PBS) containing 2% FBS. Expression of cell activation markers were examined by flow cytometry with FITC-conjugated α-CD25 antibodies and PE-conjugated α-CD69 antibodies (BD PharMingen). Propidium iodide staining was performed using a Cellular DNA Flow Cytometric Analysis Kit (Roche Molecular Biochemical) and cell cycle analyses were carried out by flow cytomertry.

### Cell extracts, immunoblotting, and immunoprecipitation

Cells were collected 48 hours after DMSO, PHA (10 ng/ml) or prostratin treatment, washed with PBS, and lysed with EBCD buffer (50 mM Tris-HCl [pH 8.0], 120 mM NaCl, 0.5% NP-40, 5 mM dithiothreitol) containing a protease inhibitor cocktail (Sigma) and a ribonuclease inhibitor (20 U/ml, Invitrogen). Total protein concentrations were determined by the Bio-Rad protein assay, and 25 μg of total protein was analyzed by sodium dodecyl sulfate (SDS)-polyacrylamide gel electrophoresis. Immunoblotting was performed as described previously using enhanced chemiluminescence (ECL) Western Blotting Substrate (Pierce) for detection [52]. Antibodies against Cyclin T1 and CDK9 were purchased from Santa Cruz Biotechnology, antibodies against HEXIM1 were kindly provided by Dr. Jiemin Wong (Baylor College of Medicine), and antibodies for β-actin were purchased from Sigma. Immunoprecipitations were carried out as previously described [11] using α-CDK9 antibodies. In some experiments, a portion of immunoprecipitates were examined in immunoblots and the remaining products were used for CTD kinase assays or Northern blots. The band intensity on immunoblots was quantified using the Personal Densitometer SI (Molecular Dynamics).

### CTD kinase assay

CTD kinase assay was performed as described [53]. Briefly, immunoprecipitates obtained as described above were incubated with a kinase reaction mixture (50 mM Tris-HCl [pH 7.4], 5 mM MgCl_2_, 2.5 mM MnCl_2_, 5 mM dithiothreitol, 5 μM ATP, 5 μCi of [γ-^32^P]-ATP [3000 Ci/mmol], and 200 ng GST-CTD substrate) at room temperature for one hour. Reaction products were analyzed on a 9% SDS-polyacrylamide gel, and the amounts of ^32^P incorportaed into the hyperphosphorylated form of CTD (CTDo) were quantified using the Storm 860 PhosphorImager system (Molecular Dynamics).

### Northern blots

Total RNA from cell extracts with the same amount of total protein or from immunoprecipitates was isolated using TRIzol reagent (Life Technologies) and Northern blot analyses were performed as described [14]. Briefly, RNA samples were resolved on a 10% urea-polyacrylamide gel, transferred and cross-linked to a nylon membrane (Perkin Elmer Life Sciences). Hybridizations were performed with the ULTRAHyb Northern blot kit (Ambion). Oligonucleotide probes for 7SK snRNA and U1 snRNA were 5' end-labeled with a T4 polynucleotide kinase (Invitrogen) and [γ-^32^P]-ATP, and purified by passing through Sephadex G-50 (Amersham Biosciences) spin columns. Hybridization signals were quantified with a Storm 860 PhosphorImager system.

### Virus infection and luciferase assay

5 × 10^6 ^resting CD4^+ ^T cells were infected by a wild type HIV luciferase reporter virus NL4-3-Luc or a mutant virus NL4-3-Luc-Tat^- ^with a non-functional Tat protein. These viruses contain a deletion in the *env *gene, a replacement of the *nef *gene with firefly luciferase gene, and are pseudotyped with VSV-G for entry. The NL4-3-Luc-Tat^- ^virus additionally contains an *EcoR*I site inserted after proline 18 of the Tat open reading frame, which abolishes Tat functions [54]. Viruses were produced as previously described [55] and 1 ml of culture medium containing the viruses was used for each infection. After overnight incubation, cells were washed twice with PBS and cultured in complete RPMI with DMSO or prostratin in the presence or absence of flavopiridol (10 or 50 nM). Cells were harvested 48 hours after treatment, washed with PBS, and luciferase assay was performed using the Luciferase Assay System (Promega) according to manufacturer's instructions, and the luciferase activity was measured with a luminometer.

### Microarray analysis and data validation

RNA samples for microarray analysis was isolated using the Qiagen RNeasy Mini Kit from resting CD4^+ ^T cell from three independent donors cultured in the presence of prostratin or DMSO for 48 hours. Microarray analysis was carried out by the Baylor Microarray Core Facility (Baylor College of Medicine, Houston, TX) according to the protocol described at the webpage of Baylor microarray core facility [56]. Briefly, RNA quality and concentration were analyzed by an Agilent 2100 Bioanalyzer and the NanoDrop ND-1000 Spectrophotometer. RNA samples were reverse transcribed to cDNA and transcribed using T7 RNA polymerase and biotinylated ribonucleotides to generate labeled cRNA. Fragmented cRNA was hybridized to Human Genome U133 Plus 2.0 Array (Affymetrix) containing ~54,000 probe sets representing over ~47,000 transcripts. Following washing and staining, arrays were scanned by an Affymetrix Gene Chip Scanner 3000, normalized to the medium intensity, and analyzed by GeneSifter (VizX Labs), a web-based microarray analysis system. Comparison tests were performed by t-test to assign a confidence level as to whether the gene is differentially expressed. Raw P-values were adjusted by Benjamini and Hochberg (False Discovery Rate) method for multiple testing corrections. Ontology analyses were done by the GeneSifter in combination with BiNGO, a Cytoscape 2.1 plugin to determine which Gene Ontology (GO) categories are statistically over-represented in a set of genes [57,58]. BiNGO maps the predominant functional themes of a given gene set on the GO hierarchy, and outputs this mapping as a Cytoscape graph. KEGG (Kyoto Encyclopedia of Genes and Genomes) pathway reports were used to categorize genes according to their involvement in biological pathways.

Microarray data were validated by reverse transcription and quantitative real-time PCR using the Bio-Rad MyIQ single color detection system as previously described [36]. Briefly, cDNA was synthesized from RNA samples using the iScript™ cDNA synthesis kit (Bio-Rad) and quantitative real-time PCR was performed using the iQ™ SYBR Green Supermix (Bio-Rad). Primers for PCR designed by the Beacon Desinger 2.0 (Premier Biosoft) were: LKLF-F (F: forward primer) 5'-GCACCGCCACTCACACCTG-3', LKLF-R (R: reverse primer) 5'-CCGCAGCCGTCCCAGTTG-3', S100A8-F 5'-GCTAGAGACCGAGTGTCCTCAG-3', S100A8-R 5'-CTGCCACGCCCATCTTTATCAC-3', EGR1-F 5'-TGGTGCCTTTTGTGTGATGCG-3', EGR1-R 5'-GCTCAGCTCAGCCCTCTTCC-3', DEFA1-F 5'-TGCCCTCTCTGGTCACCCTG-3', DEFA1-R 5'-AGGAGAATGGCAGCAAGGATGG-3', CD69-F 5'-ACACAGAGGTCAGCAGCATGG-3', CD69-R 5'-ACCACAGAGCAGCATCCACTG-3', α-Tubulin-F 5'-CCTGACCACCCACACCACAC-3', α-Tubulin-R 5'-TCTGACTGATGAGGCGGTTGAG-3', DUSP4-F 5'-GTGCTGCGGAGGCTGCTAG-3', DUSP4-R 5'-TGAAGACGAACTGCGAGGTGG-3', Cyclin T1-A-F 5'-CACAACACGACCCAGACAATAGAC-3', Cyclin T1-A-R 5'-CCACCAGACCGAGGATTCAGATAG-3', Cyclin T1-B-F 5'-GGCGTGGACCCAGATAAAG-3, Cyclin T1-B-R 5'-CTGTGTGAAGGACTGAATCATG-3', CDK9-F 5'-AGCACCAACTCGCCCTCATC-3', CDK9-R 5'-TTCAGCCTGTCCTTCACCTTCC-3'. Analyses were done by the MyIQ software program (Bio-Rad) and the fold-changes were calculated as previously described [36].

## Competing interests

The author(s) declare that they have no competing interests.

## Authors' contributions

T-LS performed the experiments, performed the analysis of microarray data, and wrote the manuscript. AR conceived the study, participated in its design, and wrote the manuscript. All authors read and approved the final manuscript.

## Supplementary Material

Additional File 1 Additional File Figure Legends. Figure legends for Additional Files 2–4. Click here for file

Additional File 2 Validation of microarray data by quantitative real-time PCR. The data show the quantitative real-time PCR validation of prostratin microarray analysis. Click here for file

Additional File 3 GO categories in biological process of transcripts regulated by prostratin. The data show the over-represented ontology pathways in biological process in prostratin microarray analysis. Click here for file

Additional File 4 KEGG pathways that are significantly affected by prostratin treatment. The table shows the over-represented KEGG pathways in prostratin microarray analysis. Click here for file
