# Supplementary material for: Effects of prostratin on Cyclin T1/P-TEFb function and the gene expression profile in primary resting CD4+ T cells
Source: Retrovirology. 2006 Oct 2;3:66. doi: 10.1186/1742-4690-3-66 (PMC1599745; doi:10.1186/1742-4690-3-66)
Supplement: Additional File 1 — Additional File Figure Legends. Figure legends for Additional Files 2–4. [file 1742-4690-3-66-S1.doc]

**Figure Legends**

**Additional File 2. Validation of microarray data by quantitative real-time PCR.** An aliquot of RNA from the three donors for microarray analysis were reverse transcribed for quantitative real-time PCR. Primers were designed to detect representative up- or down-regulated transcripts. EGR1, DUSP4, and CD69 were selected to represent up-regulated transcripts and DEFA1, LKLF, and S100A8 were chosen to represent down-regulated ones. Fold-change was calculated as the change in transcript levels in prostratin-treated cells relative to DMSO-treated cells after normalization to α-Tubulin levels.

**Additional File 3. GO categories in biological process of transcripts regulated by prostratin.** Non-redundant (A) up- or (B) down-regulated gene lists obtained from GeneSifter was analyzed by Cytoscape using BiNGO to generate the hierarchical representation of GO categories that were over-represented in biological process. The size of each node indicates the relative number of transcripts involved in the category, and the color shows the statistical significance. For example, there were 14 up-regulated transcripts in “program cell death” with a p-value of 9.35×10^-7^, and there was one up-regulated transcript in “positive regulation of dendritic cell activation” with a p-value of 4.56×10^-2^. For down-regulated transcripts, there were 30 transcripts in “protein metabolism” with a p-value of 1.29×10^-2^, and there were 4 transcripts in “regulation of growth” with a p-value of 4.67×10^-2^.
