# Supplementary figures and images for: Effects of prostratin on Cyclin T1/P-TEFb function and the gene expression profile in primary resting CD4+ T cells
Source: Retrovirology. 2006 Oct 2;3:66. doi: 10.1186/1742-4690-3-66 (PMC1599745; doi:10.1186/1742-4690-3-66)

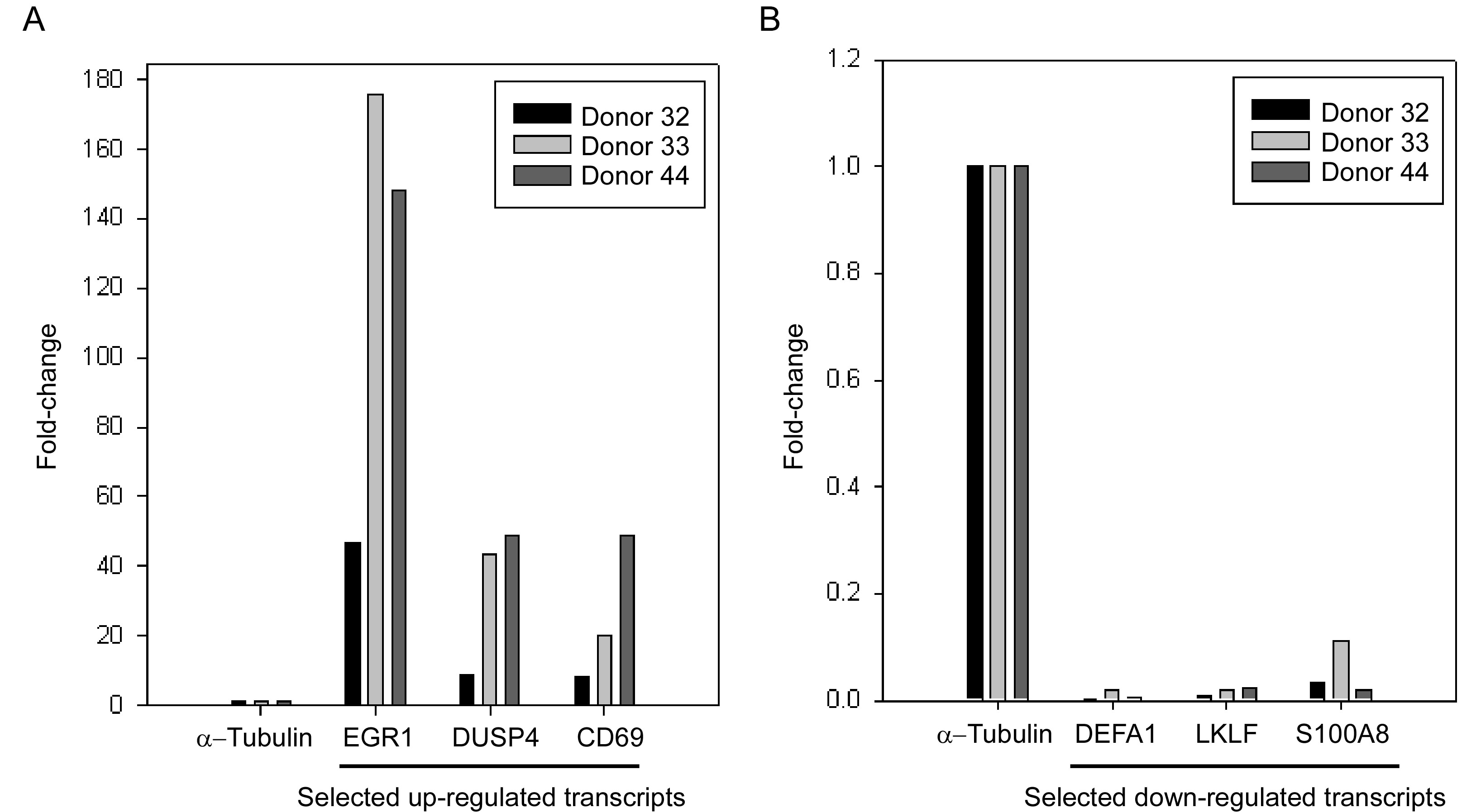

Supplement: Additional File 2 — Validation of microarray data by quantitative real-time PCR. The data show the quantitative real-time PCR validation of prostratin microarray analysis. [file 1742-4690-3-66-S2.tiff]

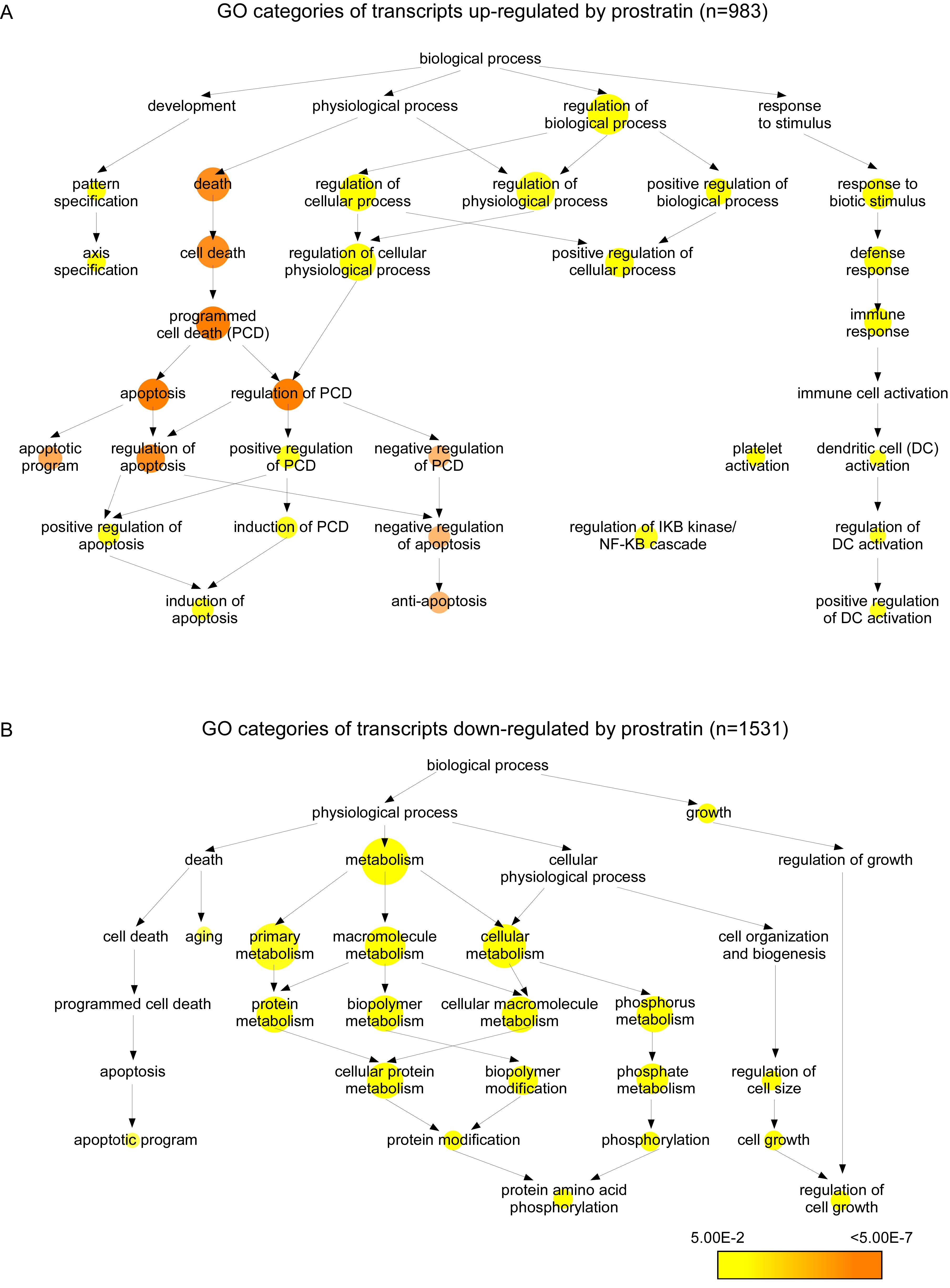

Supplement: Additional File 3 — GO categories in biological process of transcripts regulated by prostratin. The data show the over-represented ontology pathways in biological process in prostratin microarray analysis. [file 1742-4690-3-66-S3.tiff]
