# Supplementary material for: Effects of prostratin on Cyclin T1/P-TEFb function and the gene expression profile in primary resting CD4+ T cells
Source: Retrovirology. 2006 Oct 2;3:66. doi: 10.1186/1742-4690-3-66 (PMC1599745; doi:10.1186/1742-4690-3-66)
Supplement: Additional File 4 — KEGG pathways that are significantly affected by prostratin treatment. The table shows the over-represented KEGG pathways in prostratin microarray analysis. [file 1742-4690-3-66-S4.doc]

**Additional File 4**. KEGG pathways that are significantly affected by prostratin treatment

| **KEGG Pathway** | **List Up^a^** | **z-score** | **List Down^b^** | **z-score** | **Array^c^** |
| --- | --- | --- | --- | --- | --- |
| Proteasome | 13 | **7.82** |  |  | 30 |
| Apoptosis | 13 | **2.42** | 13 | **3.0** | 99 |
| Cytokine-cytokine receptor interaction | 26 | **2.05** |  |  | 256 |
| MAPK signaling pathway |  |  | 30 | **4.18** | 247 |
| Ubiquitin mediated proteolysis |  |  | 8 | **3.55** | 42 |
| Phosphatidylinositol signaling system |  |  | 10 | **3.02** | 68 |
| Wnt signaling pathway |  |  | 16 | **2.72** | 140 |

^a^ The numbers of up-regulated probes represented in genes belonging to the indicated ontology category. ^b^ The numbers of down-regulated probes represented in genes belonging to the indicated ontology category. ^c^ Total probe numbers belonging to the indicated ontology category that were present on the array.
